# Properties of the HtrA Protease From Bacterium *Helicobacter pylori* Whose Activity Is Indispensable for Growth Under Stress Conditions

**DOI:** 10.3389/fmicb.2019.00961

**Published:** 2019-05-03

**Authors:** Urszula Zarzecka, Anna Modrak-Wójcik, Donata Figaj, Malgorzata Apanowicz, Adam Lesner, Agnieszka Bzowska, Barbara Lipinska, Anna Zawilak-Pawlik, Steffen Backert, Joanna Skorko-Glonek

**Affiliations:** ^1^Department of General and Medical Biochemistry, Faculty of Biology, University of Gdańsk, Gdańsk, Poland; ^2^Division of Microbiology, Department of Biology, Friedrich-Alexander-University Erlangen-Nürnberg, Erlangen, Germany; ^3^Division of Biophysics, Faculty of Physics, Institute of Experimental Physics, University of Warsaw, Warsaw, Poland; ^4^Department of Environmental Technology, Faculty of Chemistry, University of Gdańsk, Gdańsk, Poland; ^5^Department of Microbiology, Hirszfeld Institute of Immunology and Experimental Therapy, Polish Academy of Sciences, Wrocław, Poland

**Keywords:** HtrA, *Helicobacter pylori*, virulent factor, proteolytic activity, protein quality control system, stress endurance, oligomerization

## Abstract

The protease high temperature requirement A from the gastric pathogen *Helicobacter pylori* (HtrA*_Hp_*) belongs to the well conserved family of serine proteases. HtrA*_Hp_* is an important secreted virulence factor involved in the disruption of tight and adherens junctions during infection. Very little is known about the function of HtrA*_Hp_* in the *H. pylori* cell physiology due to the lack of *htrA* knockout strains. Here, using a newly constructed Δ*htrA* mutant strain, we found that bacteria deprived of HtrA*_Hp_* showed increased sensitivity to certain types of stress, including elevated temperature, pH and osmotic shock, as well as treatment with puromycin. These data indicate that HtrA*_Hp_* plays a protective role in the *H. pylori* cell, presumably associated with maintenance of important periplasmic and outer membrane proteins. Purified HtrA*_Hp_* was shown to be very tolerant to a wide range of temperature and pH values. Remarkably, the protein exhibited a very high thermal stability with the melting point (T_m_) values of above 85°C. Moreover, HtrA*_Hp_* showed the capability to regain its active structure following treatment under denaturing conditions. Taken together, our work demonstrates that HtrA*_Hp_* is well adapted to operate under harsh conditions as an exported virulence factor, but also inside the bacterial cell as an important component of the protein quality control system in the stressed cellular envelope.

## Introduction

*Helicobacter pylori* is a microaerophilic, Gram-negative bacterium that persistently colonizes the gastric mucosa of roughly half of the world’s human population (Kusters et al., 2006; Suzuki et al., 2006). *H. pylori* infections are usually asymptomatic, however, in approximately 10–20% of infected people *H. pylori* infection is associated with the development of gastric diseases such as gastritis or peptic ulcer. In approximately 1–3% of infected individuals gastric lymphoma or gastric adenocarcinoma may develop (Bauer and Meyer, 2011). Therefore, *H. pylori* has been classified as a class I carcinogen by the WHO (IARC, 1994).

The human stomach is a hostile environment with pH of 1.5–3.5 in the lumen (Smolka and Backert, 2012). For a pathogen, successful colonization under such harsh conditions and establishment of persistent infection in the host requires the concerted action of numerous virulence factors. For example, *H. pylori* produces, among other virulence factors, urease to neutralize the acidic pH of the stomach, various adhesins to facilitate adhesion to the gastric cells, and factors required to cause epithelial barrier damage (Stingl et al., 2002; Magalhães and Reis, 2010; Rasmussen and Andersen, 2011; Kao et al., 2016). The latter group includes the HtrA*_Hp_* protein, which is involved in degradation of epithelial intercellular junction components upon infection of the host (Hoy et al., 2010; Tegtmeyer et al., 2017). HtrA*_Hp_* belongs to the well-conserved family of serine proteases, whose members are present in both prokaryotes and eukaryotes, including humans. HtrA proteins play important roles in many bacterial species, where they are typically localized in the periplasmic space and contribute to virulence of pathogens following secretion. The majority of bacterial HtrAs are involved in quality control of extracytoplasmic proteins and via their protease activity are responsible for removal of aberrant proteins from the cellular envelope (reviewed in Clausen et al., 2002). Many HtrA homologs exhibit additional chaperone-like activity to bind unfolded polypeptides and prevent these from forming undesirable interactions (reviewed in Clausen et al., 2011; Skorko-Glonek et al., 2013). Aside from these housekeeping roles, HtrAs in many pathogenic bacteria are necessary for proper maturation and export of virulence factors (Lyon and Caparon, 2004; Frees et al., 2013). For these reasons HtrAs are implicated in virulence of pathogenic *Escherichia coli, Bordetella pertussis, Borrelia burgdorferi, Chlamydia* sp., and *Salmonella enterica* serovar Typhimurium, amongst others (Frees et al., 2013; Skorko-Glonek et al., 2013; Backert et al., 2018). Extracellular HtrA of *H. pylori* strains can facilitate damage to the gastric epithelial cell layer, and its proteolytic substrates include components of the adherens junctions (E-cadherin), tight junctions (claudin-8 and occludin), and extracellular matrix proteins (e.g., fibronectin and aggrecan) (Hoy et al., 2010; Schmidt et al., 2016; Harrer et al., 2017; Tegtmeyer et al., 2017).

The HtrA protease family members contain a protease domain of a chymotrypsin type at the N-terminal part and at least one PDZ domain (post-synaptic density protein 95, *Drosophila* disc large tumor suppressor and Zonula occludens- 1 protein domain) at the C-terminus. All known HtrAs assemble into oligomers whose building units are trimers. In many cases, trimers form higher oligomeric forms (hexamers, dodecamers, or 24-mers), and oligomer transitions are usually associated with substrate binding/release (Hansen and Hilgenfeld, 2013). The best characterized member of the HtrA family is HtrA*_Ec_* (also known as DegP) from *Escherichia coli*, which is regarded as a model bacterial HtrA. This protein requires activation to perform cleavage of substrates. In the inactive state the protease active center architecture does not allow enzymatic activity, and activation requires substantial rearrangements of the HtrA molecule. This process is allosterically regulated by binding of appropriate substrates or peptides to the PDZ1 and proteolytic domain (Krojer et al., 2008a; Jiang et al., 2008; Kim and Sauer, 2012), and is accompanied with rearrangement of oligomeric forms: the resting state hexamer is converted to a spherical cage-like molecule composed of 12 or 24 subunits. After substrate cleavage is completed, the protein returns to its inactive state and the large oligomers disassemble (Kim et al., 2011). Switching between the protease inactive and active states is believed to protect cellular proteins from uncontrolled and unspecific proteolysis (Hansen and Hilgenfeld, 2013).

The active center of the HtrA*_Ec_* protease is constructed and stabilized by several regulatory loops (Clausen et al., 2002; Krojer et al., 2002). These include the L1 and L2 loops whose residues directly interact with the substrate peptide. In particular, the active site serine is located in the L1 loop, whereas the L2 loop forms an antiparallel β-sheet with the substrate peptide. Specificity at the cleaved site is determined by interactions of the substrate with the enzyme, in particular within the so-called substrate specificity pocket S1, which is formed by residues of the L1 and L2 loops (Clausen et al., 2002; Krojer et al., 2008a). Generally, HtrA*_Ec_* degrades unfolded polypeptides that expose their hydrophobic regions and preferred cleavage sites typically follow aliphatic residues (predominantly valine and isoleucine) (Jones et al., 2002). The S1 pocket of HtrA*_Ec_* is therefore lined with nonpolar residues, in particular isoleucine and alanine (Kolmar et al., 1996; Huston et al., 2011; Zarzecka et al., 2018). However, there are additional specificity determinants involving substrate sequences that bind to a special cleft in the PDZ1 domain. In consequence, efficient activation of the protease requires the simultaneous binding of a substrate peptide to the proteolytic and PDZ1 domains (Kim et al., 2011).

Compared to HtrA*_Ec_* there is considerably less information available about HtrA*_Hp_*. The majority of studies concerned the role of the extracytoplasmic fraction of this protein. Using one of its natural human substrates, E-cadherin, it was demonstrated that HtrA*_Hp_* protease activity is tolerant to high temperature (up to 65°C) and to a broad pH range (pH 4–10). Cleavage of E-cadherin was not affected by the presence of monovalent (Na^+^, K^+^) or bivalent cations (Ca^2+^, Mg^2+^, Mn^2+^) (Hoy et al., 2013). The cleavage site specificity seems to be similar to that of HtrA*_Ec_*. Using recombinant fragments of E-cadherin as a substrate it was demonstrated that HtrA*_Hp_* prefers to cut after or between hydrophobic amino acid residues (Schmidt et al., 2016). In addition, expression of HtrA*_Hp_* is induced under certain stress conditions, e.g., oxidative stress (Huang and Chiou, 2011) or low pH (Merrell et al., 2003; Tegtmeyer et al., 2016). Functional studies are carried out so far by the development and usage of both small molecule and peptide-based inhibitors (Hoy et al., 2010; Schmidt et al., 2016; Tegtmeyer et al., 2017). However, due to the lack of any *htrA_Hp_* knockout strains, the function of this protease in the *H. pylori* cell remained widely unexplored so far. Knowledge about biochemical properties of this protein is also relatively sparse to date. For these reasons we undertook efforts to fill the gaps in our understanding of cellular functions and characteristics of the HtrA*_Hp_* protein.

## Materials and Methods

### Materials

Restriction enzymes and T4 ligase were purchased from Thermo Fisher Scientific (Waltham, MA, United States). Primers used in the site-directed mutagenesis were purchased from Genomed S.A. (Warszawa, Poland). β-casein from bovine milk and lysozyme from chicken egg white, hydrogen peroxide, cumene hydroperoxide, as well as all antibiotics and other chemicals were purchased from Merck (Poznań, Poland). *Pfu* Ultra hotstart DNA polymerase was from Agilent Technologies (La Jolla, CA, United States).

### Plasmids and Strains

The strains and plasmids used in this study are listed in Table 1.

**Table 1:** Bacterial strains and plasmids.

Strain/plasmid	Genotype	References/source
DH5α	*sup*E44 Δ*lac*U169 (φ80 *lac*ZΔM1 ) *hsd*R17 *end*A1 *gyr*A96 thi-1 *rel*A1	Hanahan, 1983
*E. coli* BL21DE3	F^-^ *ompT hsdS_B_(r_B_^-^m_B_^-^) gal dcm*	Novagen
*H. pylori* 26695	Wild type strain	Tomb et al., 1997
*H. pylori* J99	Wild type strain	Alm et al., 1999
*H. pylori* N6	Wild type strain	Ferrero et al., 1992
N6 Δ*htrA*	*H. pylori* N6 *secA*R837K Δ*htrA, Kan^R^*	Zawilak-Pawlik et al., unpublished
N6 Δ*htrA/htrA_N6_*	*H. pylori* N6 *secA*R837K *ΔhtrA*/*htrA*N6, Cm^R^	Zawilak-Pawlik et al., unpublished
pJS17	pQE60, *htrA S210*A from *E. coli* with C-terminal His_6_-tag, Amp^R^	Skorko-Glonek et al., 2008
pJS18	pQE60, wt *htrA* from *E. coli* with C- terminal His_6_-tag, Amp^R^	Skorko-Glonek et al., 2003
pHJS5	pET26b, wt *htrA* from the *H. pylori* 26695 strain with C- terminal His_6_-tag, Kan^R^	This work
pHJS6	pET26b, wt *htrA* from the *H. pylori* J99 strain with C- terminal His_6_-tag, Kan^R^	This work
pUZ3	pET26b, *htrAS221A* from the *H. pylori* 26695 strain with C- terminal His_6_-tag, Kan^R^	This work
pUZ4	pET26b, *htrAS221A* from the *H. pylori* with C- terminal His_6_-tag, Kan^R^	This work
pUZN10	pET26b, wt *htrA* from the *H. pylori* N6 strain with C- terminal His_6_-tag, Kan^R^	Zawilak-Pawlik et al., unpublished
pUZN11	pET26b, *htrAS221A* from the *H. pylori* N6 strain with C- terminal His_6_-tag, Kan^R^	Zawilak-Pawlik et al., unpublished

#### Plasmid Construction

The *htrA_Hp_* genes derived from *H. pylori* strain 26695 and strain J99 were amplified by PCR using primers listed in Supplementary Table S1 and genomic DNA as a template. The amplicons were introduced into the *Nco*I and *Xho*I restriction sites of the pET26b(+) expression vector which fused the His_6_-tagged *htrA_Hp_* gene to the *E. coli pelB* signal sequence, allowing the recombinant protein to be directed to the periplasm of *E. coli*. This resulted in plasmids pHJS5 and pHJS6 (Table 1).

Subsequently, a mutation was introduced to replace the active site serine S221 (numeration of the unprocessed polypeptide) by alanine using site-directed mutagenesis according to a standard protocol of the Quick-Change mutagenesis kit (Stratagene, Agilent Technologies, Santa Clara, CA, United States) with the primers Hp_S221A_fw and Hp_S221A_rev (Supplementary Table S1). Plasmids pHJS5 and pHJS6 were used as templates, which resulted in plasmids pUZ3 and pUZ4, respectively (Table 1). Presence of the substitutions was verified by sequencing (Genomed S.A., Poland).

The *H. pylori htrA* mutant strains were constructed by gene replacement via double crossing-over recombination (Zawilak-Pawlik, unpublished). Briefly, *htrA* was replaced by *aphA-3* cassette giving an *htrA* knock-out strain. The Δ*htrA* strain was complemented by the wt *htrA*-*cat* cassette in the native *htrA* locus (construct’s schemes are presented in Supplementary Figure S1). Both strains, Δ*htrA* and Δ*htrA*/*htrA*N6, carry the same suppressor mutation in the *secA* gene (*secA*R837K), thus the differences in their phenotypes are related to the *htrA* mutation and not to any secondary level mutation effects.

### Growth Conditions

The *H. pylori* wild-type strains 26695, J99 and N6, and the N6 derivatives were grown on GC agar (Oxoid) supplemented with 10% donor horse serum (Biowest, France), protease pepton (Oxoid, Germany), 1% vitamin mix, 10 μg/ml vancomycin, 5 μg/ml trimethoprim, 8 μg/ml amphotericin, and 10 μg/ml colistin. When necessary, the media were supplemented with 10 μg/ml kanamycin or 8 μg/ml chloramphenicol. Bacteria were incubated for 2 days at 37°C under microaerobic conditions produced by a CampyGen sachet in a 2.5 L anaerobic jar (Oxoid, Germany). Colonies were harvested and suspended in BHI medium (Oxoid, Germany), bacteria were quantified by optical density (OD) at 600 nm and bacterial suspensions were normalized to OD of 0.35. To induce stress, serial dilutions of a bacterial suspension (5 μl) were spotted onto plates containing a stress-inducing agent and grown for 6 days. Puromycin (2.5 μg/ml) was used to test effects of proteotoxic stress, while the pH of the growth medium was set to 5.0, 5.5, or 8.0. After sterilization and supplementation with donor horse serum, vitamin mix and antibiotics, the pH of the broth was verified prior to plate preparation using PH 5 food tester kit (cat. number: EHX8.1, Roth, Germany). The final pH values were 5.2, 5.6, and 7.7, respectively, while standard GC agar plates were pH 7.1.

To induce osmotic shock, nonionic (175 mM sucrose) or ionic (85 mM NaCl or 32 mM MgCl_2_) osmolytes were added to the GC medium. Bacteria were incubated for 2 days at 37°C after which a heat shock was induced by increasing the temperature to 39 or 41°C. To determine sensitivity to oxidative stress a disk diffusion assay was performed. Three hundred microliter aliquots of bacterial suspensions in the BHI medium (OD_600_ 0.4) were spread over the entire surface of the GC agar plates to generate a lawn. Five microliter portions of 2% cumene hydroperoxide or 10 μl portions of 30% hydrogen peroxide were added to sterile 7 mm diameter disks (Whatman 3 mm) and placed on the top of the inoculated agar. The plates were incubated at 37°C or 39°C and after 3 days the inhibition zones were measured. All experiments were performed at least three times.

### Expression and Purification of the HtrA Proteins

*E. coli* BL21 (DE3) transformed with the appropriate plasmid was used to overproduce the wild-type or the proteolytically inactive His_6_-tagged HtrA*_Hp_*S221A variant in the pET System (Novagen, San Diego, CA, United States). Bacteria were grown at 37°C in Luria-Bertani (LB) broth supplemented with 50 μg/ml kanamycin to OD_600_ 0.6–0.7; expression of *htrA* was induced by addition of 0.5 mM isopropyl-β-D-thiogalactopyranoside (IPTG). After 2–3 h the bacteria were centrifuged (10 min, 5000 ×*g*) and the pellet was resuspended in 15–20 ml of lysis buffer BH10 (50 mM HEPES pH 8.0, 300 mM KCl, 10 mM imidazole pH 8.0). Lysis was completed by addition of lysozyme to a final concentration of 1 mg/ml followed by sonication. DNase I (5 μg/ml) was used to remove DNA and all lysates were cleared by centrifugation at 25,000 ×*g* for 30 min at 4°C. The HtrA proteins were purified by nickel-affinity chromatography under native or denaturing conditions, according to the manufacturer‘s instructions (Ni-NTA, Qiagen, Germany) with some modifications as described in Zarzecka et al. (2018). In the case of purification under denaturing conditions one further modification was introduced: the proteins that precipitated during dialysis (not the supernatant) were dissolved in 50 mM HEPES, 8 M Urea, pH 8.0 and submitted to Ni-NTA chromatography. For analytical centrifugation the elution buffer was exchanged with 50 mM sodium phosphate, 300 mM NaCl, pH 6.5, using Amicon Ultra 30K microconcentrators. The wild-type (wt) and S210A HtrA*_Ec_* variants were expressed and purified exactly as described in Zarzecka et al. (2018).

### Analysis of the Proteolytic Activity

To analyze the activity of HtrA*_Hp_* and HtrA*_Ec_* toward unfolded versus folded substrates, β-casein and native or chemically reduced lysozyme were used (Huston et al., 2011; Marsh et al., 2013). The proteolytically active or inactive variants of HtrA (0.52 μM) were incubated for 90 min at 37°C with 21 μM β-casein or 35 μM lysozyme in reaction buffer (50 mM HEPES, 200 mM NaCl, pH 6.2), in a final volume of 200 μl. The reactions with lysozyme were performed with or without 7 mM Tris (2-carboxyethyl) phosphine hydrochloride (TCEP) as a reductant. Samples without HtrA were used as controls. The reaction was terminated by the addition of Laemmli lysis buffer (30 mM Tris- HCl, pH 6.8, 5% glycerol, 1.5% sodium dodecyl sulfate, 0.005% bromophenol blue) and immediate freezing at -20°C (Figaj et al., 2014). The samples were then resolved in 15% SDS-PAGE and the gels were stained with Coomassie Brilliant Blue. The gels were analyzed densitometrically using a 1DScan EX program (Scanalytics Inc., United States).

To determine the temperature dependence of the apparent cleavage rates of HtrA*_Hp_* and HtrA*_Ec_* the proteolytically active HtrA variants (0.17 μM) were incubated at temperatures in the range of 25–80°C with 17 μM β-casein in reaction buffer. For pH dependence at 37°C a set of buffers was used in combination with 100 mM NaCl: 50 mM HEPES at pH 6.0, 6.5, 7.0, 7.5, or 8.0; 50 mM Na-acetate at pH 5.0 or 5.5; and 50 mM Na-carbonate at pH 8.5, 9.0, 9.5, and 10.0. The final volume of the reaction mixture was 250 μl. At certain time points samples were withdrawn in triplicates, mixed with Laemmli lysis buffer and frozen prior to gel electrophoresis.

The cleavage site specificity of HtrA*_Hp_* was determined as described in Zarzecka et al. (2018). In brief, 0.17 μM HtrA*_Hp_* was mixed with 10 μM β-casein in 50 mM HEPES pH 6.5, 100 mM NaCl or 0.25 μM HtrA*_Hp_* with 9 μM lysozyme in 50 mM HEPES pH 6.5, 100 mM NaCl, 0.35 mM TCEP, and incubated at 35°C. Samples (200 μl) were withdrawn every 2 min (for β-casein) or every 30 min (for lysozyme). The resulting cleavage products were identified via mass spectrometry using an LC-MS (ESI) MS-Bruker Daltonics HCT Ultra coupled with LC-Agilent Technologies 1200 series as described in Glaza et al. (2015). A digestion pattern and sequence search was performed using the Mascot data base. The peptide sequences were identified based on the m/z signals. The HtrA*_Hp_* self-cleavage products were used as a control and the identified peptides were not included in the final analysis.

### Size Exclusion Chromatography (SEC)

SEC was performed as described in Zarzecka et al. (2018) adapted from Krojer et al. (2010), Glaza et al. (2015), and Iwanczyk et al. (2007) on a Superose 12HR 10/30 column (GE Healthcare Life Sciences) equilibrated with 300 mM NaCl, 50 mM Na-phosphate, pH 6.5 or 8.0, as specified. Protein samples (50 μl), containing a refolded HtrA variant (41 μM) with or without 41 μM β-casein as a substrate, were analyzed at room temperature at a flow rate of 0.3 ml/min using an Agilent High Performance Liquid Chromatography (HPLC) system. Mixtures of HtrA with the substrate were pre-incubated for 10 min at 30 or 37°C prior to loading onto the column. The calibration standards included thyroglobulin (669 kDa), apoferritin (443 kDa), β- amylase (200 kDa), alcohol dehydrogenase (141 kDa), bovine serum albumin (67 kDa), and carbonic anhydrase (29 kDa) (Sigma-Aldrich). The protein fractions were collected and resolved by 15% SDS-PAGE.

### Analytical Ultracentrifugation

Sedimentation velocity experiments were performed at 25°C using a Beckman Optima XL-I analytical ultracentrifuge with the four-position An-Ti rotor using interference and absorption detection at 280 nm. Three hundred and ninety microliter of the refolded HtrA samples and 400 μl of a reference buffer (50 mM phosphate pH 6.5, 300 mM NaCl) were loaded into the right and the left sector, respectively, of a double-sector 1.2 cm cells with charcoal-filled epon centerpieces and sapphire windows. The samples contained proteolytically inactive HtrA*_Hp_*S221A variants at concentrations of 0.6–3.3 mg/ml. Protein concentration was determined spectrophotometrically (Jasco V-650 spectrophotometer) from absorption values at 280 nm using the extinction coefficients 𝜀^0.1%^ = 0.373 and 0.372 cm^-1^ (mg/ml)^-1^ calculated from the amino acid composition by ProtParam (Gasteiger et al., 2005) for the protein of strains 26695 and N6, respectively. Sedimentation velocity experiments were recorded at 40,000 rpm and radial absorption scans of protein-concentration profiles were measured at 4 min intervals. The data were analyzed using the SEDFIT program with the continuous sedimentation coefficient distribution *c*(*s*) model based on the Lamm equation (Schuck, 2000). Integration of the *c*(*s*) peaks provided the signal weighted average sedimentation coefficients (*s*). The partial specific volume of HtrA*_Hp_* (as determined from its amino acid composition) as well as the density and viscosity of the buffer were calculated using the Sednterp program (Laue et al., 1992).

### Circular Dichroism Measurements

Far-UV circular dichroism (CD) of the HtrAS221A preparations (0.4 mg/ml in 10 mM Na-phosphate, 100 mM NaCl, at pH 5.5, 6.5, or 8.0) was measured at a temperature range of 25–90°C in steps of 2.5°C and signals at 218 nm were recorded. The measurements were performed in cells with a 1 mm path length using a spectropolarimeter (JASCO J-815, Japan) as described before (Zurawa-Janicka et al., 2013; Jarzab et al., 2016). The melting point temperature (T_m_) and errors (SD) were calculated from the experimental data as described in Figaj et al. (2016).

### Casein Zymography

Bacterial cell pellets or purified proteins were suspended in phosphate buffer saline (PBS) and added to Laemmli buffer. The samples were loaded onto 10% SDS- PAGE gels containing 0.1% casein (Carl Roth, Germany) and separated under nonreducing conditions. Subsequently, in-gel proteins were renatured by incubation of the gel in 2.5% Triton X-100 solution at room temperature for 60 min with gentle agitation and equilibrated overnight in the developing buffer (50 mM Tris-HCl, pH 7.4, 200 mM NaCl, 5 mM CaCl_2_, 0.02% Brij35) at 37°C (Boehm et al., 2012, 2015; Albrecht et al., 2018). Transparent bands of proteins with caseinolytic activity were visualized by staining with 0.5% Coomassie Blue R250 as described (Löwer et al., 2008).

### Sequence Analysis

The following protein sequences were extracted from the UniProt database: HtrA*_Hp_* from *H. pylori* strain 26695 and J99 strains (G2J5T2 and Q9ZM18, respectively), HtrA/DegP and DegQ from *E. coli* K12 (P0C0V0 and P39099, respectively), and HtrA*_Cj_* from *C. jejuni* strain NCTC 11168 (Q0P928). The sequence of HtrA*_Hp_* from *H. pylori* N6 strain was derived from the genome sequence (Zawilak-Pawlik et al., unpublished). Sequence alignments were performed using ClustalX software.

## Results

### Effect of the Lack of htrA on Survival of *H. pylori* Under Stress Conditions

A direct analysis of the functions that HtrA*_Hp_* plays in the bacterial cell was previously hampered by a lack of HtrA-deficient *H. pylori* strains. A search comprising over 100 strains originating from a wide geographic range eventually resulted in strain N6 in which an *htrA* knockout could be established. The wild-type strain N6 and its derivative N6 Δ*htrA*/*htrA*_N6_ (complementation strain) showed similar levels of HtrA*_Hp_* expression with comparable caseinolytic activity in acrylamide gels of N6 and N6 Δ*htrA*/*htrA*_N6_ protein extracts (Zawilak-Pawlik et al., unpublished). Hence, we considered this expression system suitable for a comparative analysis of the Δ*htrA* phenotype of *H. pylori* grown under physiological and stress conditions.

**FIGURE 1:**
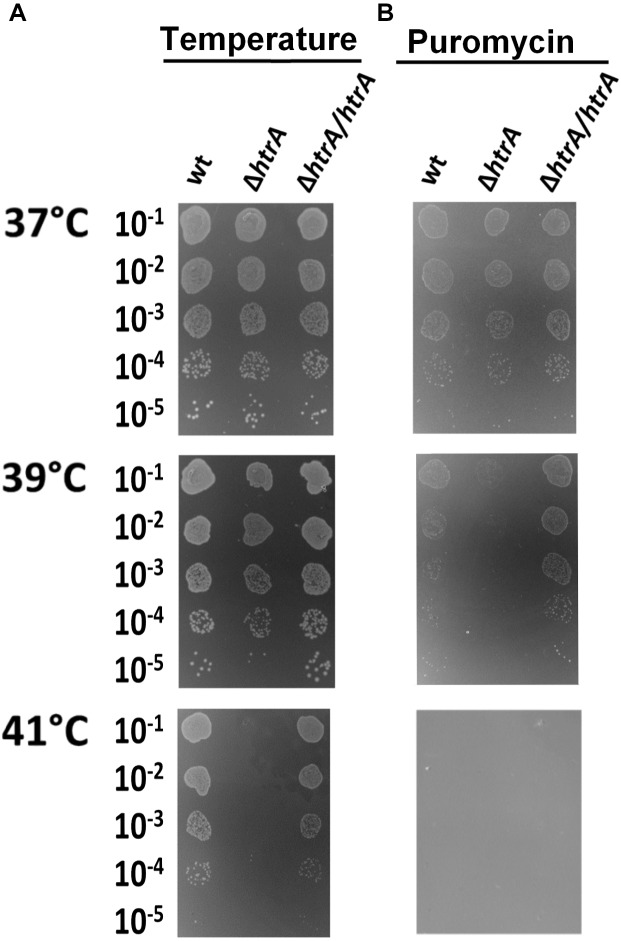
Effects of temperature **(A)** and puromycin **(B)** on growth of *H. pylori* N6 and its *ΔhtrA* derivative. Serial dilutions of bacterial cultures were spotted onto GC agar plates without **(A)** and with puromycin **(B)**, and bacterial growth at three temperatures was assessed after 6 days of incubation. The experiments were performed in triplicate and representative examples are shown.

When *H. pylori*Δ*htrA* was grown under nonstress conditions (37°C, standard GC growth medium) no visible growth defects were apparent, as it grew equally well compared to its isogenic wt strain (Figure 1A and Supplementary Figure S2A, upper panels). However, under stress conditions, the lack of HtrA*_Hp_* affected growth significantly. First, *H. pylori*Δ*htrA* showed reduced growth at elevated temperatures. At 39°C, the deletion mutant grew slower (as judged from the size of colonies) and at 41°C no growth was visible at all. As control, genetic complementation of the knockout mutation with wt *htrA* (Δ*htrA/htrA_Hp_*) restored growth capacity at all tested temperatures (Figure 1A and Supplementary Figure S2A). The second stress situation was simulated by addition of puromycin. This antibiotic causes premature release of polypeptide chains from ribosomes (Blobel and Sabatini, 1971), which leads to accumulation of misfolded or aberrant proteins (Salomons et al., 2009). Exposure to puromycin affected all tested *H. pylori* strains, especially when combined with an elevated temperature of 39°C, but growth was particularly reduced in the Δ*htrA* mutant. Compared to wt, growth of N6 Δ*htrA* at 39°C was at least three logs lower when puromycin was present (Figure 1B, middle panel, and Supplementary Figure S2B). A temperature of 41°C was fully lethal in presence of puromycin for all tested *H. pylori* strains (Figure 1B, lower panel). Interestingly, the Δ*htrA* knockout strain did not show increased sensitivity to the presence of oxidants and exposure to H_2_O_2_ or cumene hydroperoxide; all oxidants affected the Δ*htrA* mutant and the control strains similarly (Supplementary Figure S3).

**FIGURE 2:**
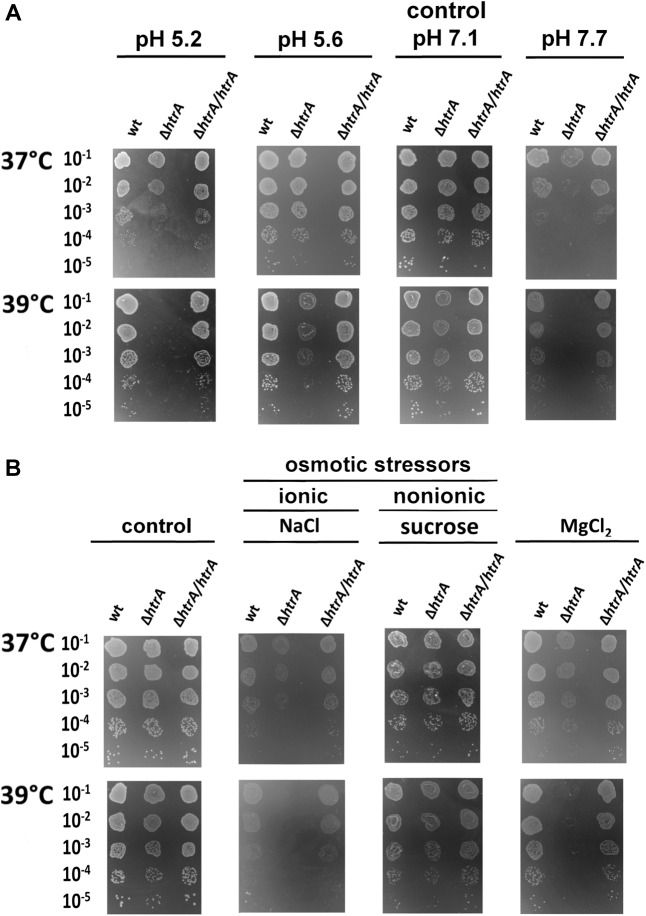
Effects of pH **(A)** and osmotic stress **(B)** on growth of *H. pylori* N6 and its *ΔhtrA* mutant. For osmotic stress, bacteria were grown for 6 days in the presence of sucrose, NaCl or MgCl_2_ at 37 or 39°C. The experiments were performed in triplicate and the representative examples are shown.

Deletion of *htrA* also rendered *H. pylori* more sensitive to pH changes. Even small pH shifts, e.g., increase of pH 7.1–7.7 or a decrease to 5.6 resulted in a reduction of growth, especially at the elevated temperature of 39°C (Figure 2A and Supplementary Figure S2C). In contrast, exposure to osmotic stress caused by a nonionic osmoticum, in this case sucrose (175 mOsm), had no significant effect on any of the tested strains (Supplementary Figure S2D, middle panels). However, an ionic osmoticum (NaCl) largely restricted growth of the bacteria, especially at 39°C. The effect of salt stress was much stronger when *htrA* was deleted, where at 37°C it reduced growth by at least one log and the colonies were markedly smaller, while at 39°C no growth was visible. Complementation with *htrA* restored the ability to grow at both temperatures. The deletion mutant also showed an increased sensitivity to presence of MgCl_2_, while no adverse effects were observed for wild-type or complemented bacteria. At 39°C the deletion mutant did not grow in presence of MgCl_2_ (Figure 2B and Supplementary Figure S2D).

### Engineering and Heterologous Expression of htrA*_Hp_* Gene Variants

**FIGURE 3:**
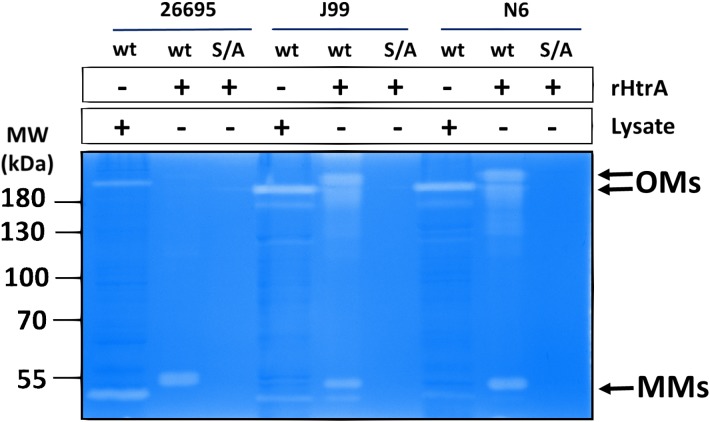
Proteolytic activity of purified recombinant HtrA*_Hp_* proteins and HtrA*_Hp_* from the *H. pylori* cell lysates. The caseinolytic activity of HtrA*_Hp_* from three indicated *H. pylori* strains was analyzed by polyacrylamide gel electrophoresis. The activity was detected in the purified recombinant wild- type (wt), but not in the corresponding S221A (S/A) HtrA*_Hp_* proteins (100 ng). HtrA*_Hp_* activities from *H. pylori* cell lysates (26695, J99, and N6 wild-type strains) are shown as control. The ability to cleave β-casein was analyzed by casein zymography. Positions of the proteolytically active HtrA*_Hp_* monomers (MMs) and oligomers (OMs) are indicated with arrows. The recombinant HtrA*_Hp_* variants migrated in gels at a higher position than their normal molecular weight due to presence of the His_6_-tag. The experiments were performed in triplicate and the representative example is shown.

The biochemical properties of HtrA*_Hp_* were further investigated using recombinant protein variants expressed in *E. coli.* For this, we constructed plasmids, coding for HtrA*_Hp_* fused with the *E. coli* PelB signal sequence at the N- terminus to ensure membrane trafficking and with a His-tag at the C- terminal that allowed purification. Three HtrA*_Hp_* proteins, showing certain differences in the amino acid composition (Supplementary Figure S4), were compared, from *H. pylori* strains 26695, J99 and N6. 26695 and J99 are well characterized and frequently used as model *H. pylori* strains (Alm et al., 1999), while strain N6 strain was included as it allowed deletion of the *htrA* gene (Tegtmeyer et al., 2016; Zawilak-Pawlik et al., unpublished). For structural studies, we generated mutants in which the crucial serine of the active site was replaced by alanine (HtrA*_Hp_*S221A) which rendered the protein proteolytically inactive (Figure 3). A corresponding S210A mutant of HtrA*_Ec_* (HtrA*_Ec_*S210A) was included as a control. The S221A variants were produced for each of the three strains and heterologously expressed in *E. coli*. The Ni^2+^-affinity chromatography was used to purify the recombinant proteins either under native or denaturing conditions with subsequent refolding. This latter method allowed the removal of the HtrA-bound ligands (data not shown).

To compare the caseinolytic activity of the purified recombinant HtrA*_Hp_* preparations with that of the native *H. pylori* enzymes, zymography was performed (Figure 3). As expected, recombinant HtrA*_Hp_* had proteolytic activity toward casein, but the S221A variants were unable to digest casein. Interestingly, analysis of the zymogram revealed that the HtrA*_Hp_* oligomers were remarkably stable and in part could even withstand denaturing conditions of the gel electrophoresis (including presence of 1.5% SDS). This ability seemed to be strain-dependent, as HtrA*_Hp_* from 26695 had a weaker ability to retain oligomers than that from strains J99 or N6.

### Characterization of the Proteolytic Activity of the HtrA*_Hp_* Protein Toward Model Substrates

Proteolytic properties of HtrA*_Hp_* were tested using two model substrates: β-casein, which is naturally unstructured (Creamer et al., 1981) and regarded as a universal protease substrate, and two structural forms of lysozyme, tested in native form and chemically denatured by reduction. These substrates were previously used to characterize other bacterial HtrAs, including HtrA*_Ec_* from *E. coli* (reviewed in Skorko-Glonek et al., 2017). The substrates were used to perform a comparative analysis of the catalytical properties of HtrA*_Hp_*_._

**FIGURE 4:**
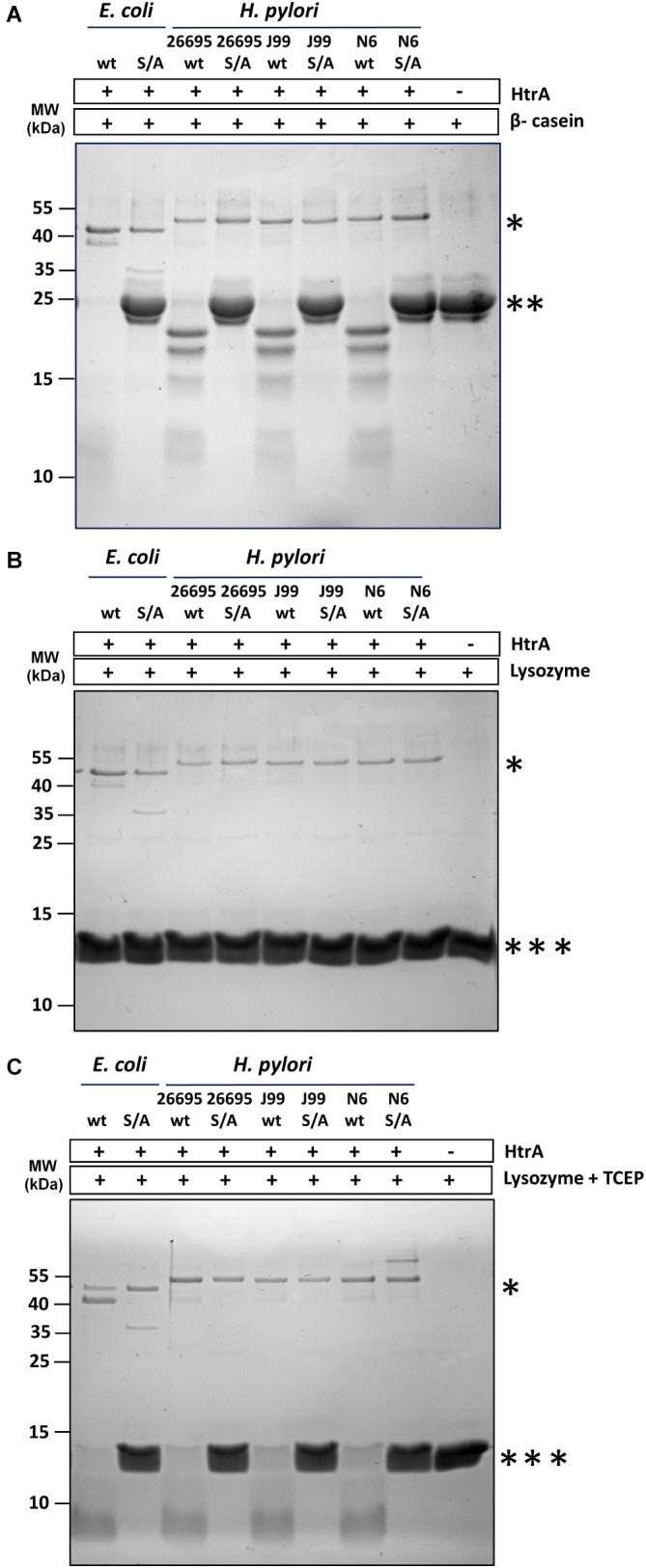
HtrA*_Hp_* exclusively degrades unfolded or denatured proteins. The proteolytically active and inactive variants HtrA*_Ec_*S210A (S/A) and HtrA*_Hp_*S221A (S/A) originating from the 26695, J99, and N6 strains were incubated with **(A)** β-casein or **(B,C)** lysozyme in 50 mM HEPES pH 6.2, 200 mM NaCl at 37°C for 90 min. The reactions with lysozyme were performed without **(B)** or with **(C)** 7 mM TCEP. Samples without HtrA were used as controls. The molar ratio of HtrA/β-casein was 1:28; HtrA/lysozyme was 1:47. Proteins were resolved in 15% gels and stained with Coomassie Brilliant Blue. All assays were performed at least three times and examples of the representative electrophoregrams are shown. The asterisks indicate: ^∗^ HtrA, ^∗∗^ β-casein, and ^∗∗∗^ lysozyme.

All three tested wt HtrA*_Hp_* (originating from strains 26695, J99 and N6) had proteolytic activity toward β-casein (Figure 4A), and against denatured lysozyme (Figure 4C), but native lysozyme was not degraded (Figure 4B). The S221A mutants displayed no proteolytic activity under the same conditions, which confirmed that the observed degradation process was not mediated by impurities in the enzyme preparations.

**FIGURE 5:**
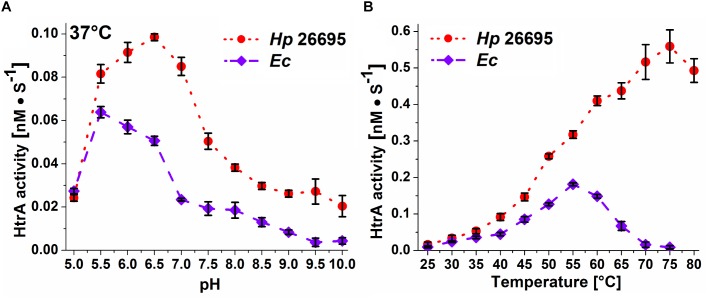
Dependence of the proteolytic activity of recombinant HtrA*_Hp_* 26695 and HtrA*_Ec_* on **(A)** pH, and **(B)** temperature. The pH-dependence was measured at 37°C, while the temperature-dependence was measured at pH 6.2. The error bars represent standard deviation values from three independent measurements.

β-casein was used to determine activity profiles of HtrA*_Hp_* at 37 and 42°C for a pH range of 5–10. The enzyme had the highest proteolytic activity between pH 5.5 and 7.0 with a maximum at pH 6.0–6.5. In comparison, HtrA*_Ec_* had its optimum at a slightly lower pH of 5.5 (Figure 5A and Supplementary Figure S5).

The effect of temperature on the proteolytic activity of HtrA*_Hp_* was assayed at the range of 25–80°C. A pH value of 6.2 was chosen for these experiments as it is considered to be the pH of the *H. pylori* periplasm where HtrA*_Hp_* is located (Rektorschek et al., 1998). At temperatures up to 40°C the apparent cleavage rates of HtrA*_Hp_* and HtrA*_Ec_* were comparable; at higher temperatures HtrA*_Hp_* degraded β-casein much more efficiently than HtrA*_Ec_* did, with a maximum activity at 70–75°C. HtrA*_Ec_* reached its maximal proteolytic activity at 55° which is consistent with previous studies (Skorko-Glonek et al., 1997) and above 70°C HtrA*_Ec_* activity was hardly detectable (Figure 5B).

### Thermal Stability of the HtrA*_Hp_* Protein

To explain the exceptionally strong activity of the HtrA*_Hp_* protein at high temperatures, we measured the thermal stability of the protein at three pH values using CD. Analysis of the CD spectra in the far UV region (170–260 nm) collected at a temperature range of 50–95°C allowed us to follow the denaturation pattern of the protein. Rising the temperature above a critical value typically leads to protein unfolding and subsequent loss of protein secondary structures. These changes are reflected by changes in the CD spectra (Kelly and Price, 2000) and it allows to calculate the protein melting temperature (T_m_) at which both folded and unfolded states of a protein are present in equal fractions. The T_m_ value serves as a determinant of protein stability. For these calculations we used CD values measured at 218 nm, as this wavelength corresponds to the minimum of the CD signal typical for β-sheets (Kelly and Price, 2000). These structures are abundant in HtrAs, especially in the proteolytic domain which is composed of two β-barrels (Clausen et al., 2011). We found that the T_m_ values of HtrA*_Hp_* exceeded 85°C (Table 2) and were markedly higher than those of HtrA*_Ec_* (by app. 14°C at pH 6.5) which corresponded well with the determined temperature preferences of these proteases. Thermal stability of these proteins did not seem to be affected by pH, as the T_m_ values at pH 5.5, 6.5, or 8.0 calculated for HtrA*_Hp_* (Table 2) and HtrA*_Ec_* (Zarzecka et al., 2018) were similar and did not differ by more than 5°C.

**Table 2:** Thermal stability of the HtrA*_Hp_*S221A (26695 strain) protein at various pH conditions.

	HtrA*_Hp_*S221A
**pH**	**T_m_ (°C)**
5.5	85.72 ± 0.24
6.5	87.50 ± 0.39
8.0	88.77 ± 0.30

### Cleavage Site Specificity

**FIGURE 6:**
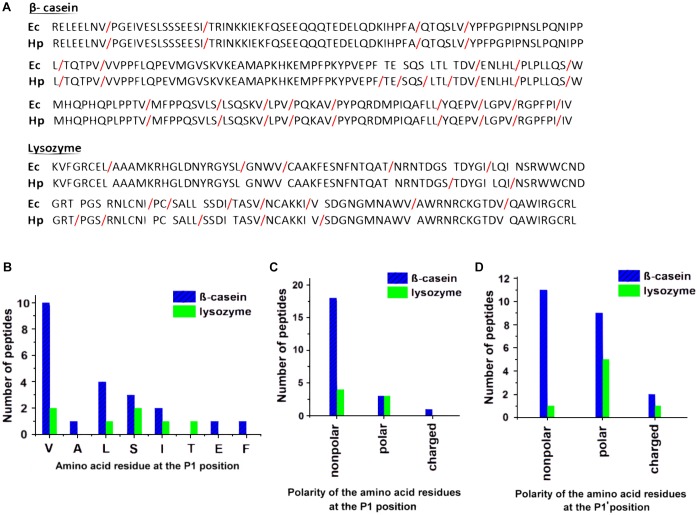
Cleavage site specificity of HtrA*_Hp_*. **(**A) Comparison of the HtrA*_Hp_* 26695 and HtrA*_Ec_* cleavage sites identified in the initial digestion products of β-casein and lysozyme using LC-MS. Red slashes indicate cleavage sites. The positions of the bonds cut by HtrA*_Ec_* are according to Zarzecka et al. (2018). **(B–D)** Analysis of the primary specificity of HtrA*_Hp_* based on digestion products of β-casein and lysozyme. The P1 **(B,C)** and P1’ **(D)** occurrences were plotted against the type of amino acid that was identified at these positions.

To expand our knowledge on the cleavage site specificity of HtrA*_Hp_* and compare it with those of other HtrA homologs examined thus far, we analyzed the most abundant peptide products generated by HtrA*_Hp_*-mediated proteolytic cleavage of β-casein and reduced lysozyme. This enabled us to determine the amino acid sequences recognized and cleaved by HtrA*_Hp_*. The identified cleavage sites are shown in Figure 6A and summarized in Table 3, whereas frequencies of amino acids occurring at the P1 (preceding the cleavage site) or the P1’ (following the cleavage site) positions of the cleaved peptides are shown in Figure 6B–D.

**Table 3:** HtrA*_Hp_* cleavage sites identified in β-casein and lysozyme.

	HtrA*_Hp_*							
	P4	P3	P2	P1	P1’	P2’	P3’	P4’
	E	L	N	V	P	G	E	I
	H	P	F	A	Q	T	Q	S
	Q	S	L	V	Y	P	F	P
	I	P	P	L	T	Q	T	P
	Q	T	P	V	V	V	P	P
	V	E	P	F	T	E	S	Q
	E	S	Q	S	L	T	L	T
	N	L	H	L	P	L	P	L
**β-casein**	L	L	Q	S	W	M	H	Q
	S	V	L	S	L	S	Q	S
	Q	S	K	V	L	P	V	P
	A	F	L	L	Y	Q	E	P
	Q	E	P	V	L	G	P	V
	P	F	T	E	S	Q	S	L
	S	L	T	L	T	D	V	E
	L	G	P	V	R	G	P	F
	P	F	P	I	I	V		
	Q	K	A	V	P	Y	P	Q
	P	P	T	V	M	F	P	P

	D	G	R	T	P	G	S	R
	T	A	S	V	N	C	A	K
	S	A	L	L	S	S	D	I
**Lysozyme**	K	K	I	V	S	D	G	N
	I	L	Q	I	N	R	S	W
	T	P	G	S	R	N	L	C
	T	D	G	S	T	D	Y	G

HtrA*_Hp_* cuts preferentially after valine at P1, with serine, leucine and isoleucine at decreasing order of preference. At P1’ HtrA*_Hp_* could accommodate a greater variety of residues with almost identical preference for nonpolar or polar/charged moieties. Hence, the overall specificity of HtrA*_Hp_* for the prime sites flanking the scissile bond was very similar to that of HtrA*_Ec_* (Zarzecka et al., 2018). Surprisingly, localization of cleavage sites of these proteases within the substrates showed significant differences, in particular as observed with lysozyme. This result suggests that the two enzymes differ in their specificities at the nonprime sites.

### Quaternary Structure of the HtrA*_Hp_* Protein

It is well established that bacterial HtrAs form oligomers with a trimer as the basic building unit, and in many cases trimers assemble into higher order oligomers (Hansen and Hilgenfeld, 2013). The quaternary structure of HtrA*_Hp_* in solution has not been characterized in detail thus far, although crystallographic data confirmed that this protein assembles into hexamers (Perna et al., 2014). As judged from the zymography assays, HtrA*_Hp_* was able to form higher order oligomers (Hoy et al., 2012 and Figure 3), however, their size was indefinable by this method.

**FIGURE 7:**
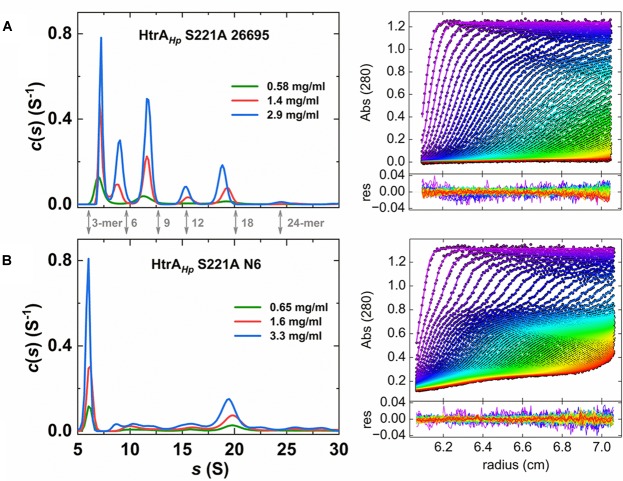
Sedimentation velocity data for proteolytically inactive S221A mutants of HtrA*_Hp_* from the *H. pylori* strains 26695 **(A)** and N6 **(B).** Left panel: sedimentation coefficient distributions *c*(*s*) for different concentrations of HtrA*_Hp_*. Theoretical *s*-values of HtrA*_Hp_ n*-mers are indicated by gray arrows (see also Table 5). Sedimentation velocity data observed at 280 nm were analyzed with a continuous sedimentation coefficient distribution *c*(*s*) model. Right panel: experimental data for the distribution shown in the left panel for HtrA*_Hp_* samples of the highest concentration (∙) with the best fits of SEDFIT *c*(*s*) model (—). Bottom panels present the fitting residuals. Centrifugation was performed at 40,000 rpm and 25°C. Radial profiles were measured at 4 min intervals in 50 mM phosphate pH 6.5, 300 mM NaCl.

Moreover, the HtrA*_Hp_* preparations from various strains showed different patterns of oligomeric forms in gels. To dispel doubts about the oligomerization status of this protein we performed sedimentation velocity ultracentrifugation experiments using preparations of refolded, proteolytically inactive proteins. We analyzed the properties of the two S221A variants of strains 26695 and N6, which produced different zymography patterns (Figure 3) and therefore were expected to form different oligomeric forms or exhibit differences in oligomer stability. We found that both HtrA*_Hp_* S221A variants sedimented as a mixture of various oligomeric forms, with sedimentation coefficients in the range of 6 or 7 S to 25 or 26 S (Table 4 and Figure 7), which correspond to trimers up to 24-mers (Table 5). However, the contribution of individual fractions was markedly different in the two HtrA*_Hp_* S221A variants. Preparations based on HtrA*_Hp_* from strain 26695 contained three major species (in combination representing approximately 80% of the total protein) with 7 S, 9 S, and 11.5 S, most likely corresponding to trimers, hexamers and nonamers, respectively. Small quantities of larger oligomers were detected at 15.5 S (12-mer), 19.0 S (18-mer, approximately 14%), and 25 S (24-mer) (Figure 7 and Table 4). The content of smaller oligomers (from trimers to nonamers) depended on the protein concentration in the sample. The peak positions did not fully correspond to the theoretical *s*-values of the HtrA*_Hp_* oligomers (see Tables 4, 5). According to Schuck (2003) this would indicate that, at least for HtrA*_Hp_* of the 26695 strain, trimers, hexamers and nonamers are in dynamic equilibrium in solution. Different results were obtained with HtrA*_Hp_* from strain N6. Two major species sedimented at about 6 S (38% of the total) and 20 S (30%) which correspond to trimers and 18-mers, respectively. Presence of small amounts (1–8%) of other oligomers (from hexamers to larger particles up to 40 S) was also detected (Table 4), while the content of the oligomeric forms did not depend on protein concentration. Hence, proteolytically inactive HtrA*_Hp_* of strain N6 exists as a mixture of multiple stable oligomeric forms, among which trimers and 18-mers dominate.

**Table 4:** Sedimentation coefficients of the HtrA*_Hp_*S221A variants calculated from *c*(*s*) distributions (see Figure 7).

Concentration (mg/ml)	s [S] (content %)							
	Peak 1	Peak 2	Peak 3	Peak 4	Peak 5	Peak 6	Peak 7	Peak 8
	**HtrA*_Hp_*S221A 26695**							
0.58	7.1 ± 0.5	–	11.3 ± 0.9	–	15.5 ± 0.8	19.3 ± 1.1	–	24.8 ± 1.2
	(50)		(29)		(4.5)	(14)		(1.7)
1.4	7.2 ± 0.3	8.8 ± 0.4	11.6 ± 0.4	–	15.6 ± 0.5	19.2 ± 0.5	–	25.7 ± 0.9
	(35)	(14)	(30)		(6.2)	(13)		(1.3)
2.9	7.3 ± 0.2	9.0 ± 0.4	11.7 ± 0.4	–	15.4 ± 0.4	18.8 ± 0.4	–	24.5 ± 0.7
	(24)	(20)	(33)		(6.4)	(14)		(1.4)
	**HtrA*_Hp_*S221A N6**							
0.65	6.1 ± 0.3	–	11.0 ± 1.1	–	15.8 ± 0.8	19.9 ± 1.1	–	24.5 ± 1.0
	(38)		(12)		(8.1)	(29)		(4.8)
1.6	6.1 ± 0.3	–	10.3 ± 0.8	13.0 ± 0.6	15.8 ± 0.9	19.9 ± 1.1	–	25.6 ± 1.2
	(38)		(8.8)	(3.2)	(7.9)	(30)		(5.9)
3.3	6.1 ± 0.2	8.9 ± 0.4	10.6 ± 0.5	12.4 ± 0.6	15.7 ± 1.0	19.5 ± 0.8	22.8 ± 0.6	25.8 ± 0.8
	(38)	(3.4)	(5.1)	(3.8)	(9.0)	(28)	(3.1)	(3.6)

**Table 5:** Theoretical *s*-values of HtrA*_Hp_ n*-mers^a^.

*n* =	1	3	6	9	12	15	18	21	24
	2.9	**6.1**	9.7	12.7	15.4	17.8	20.1	22.3	24.4

*^a^Theoretical values of sedimentation coefficients of various oligomeric forms of HtrA from H. pylori calculated according to the formula: s_n_ = s_1_ ⋅ n^2/3^ (Schuck et al., 2015), assuming that the sedimentation coefficient of the trimer is s_3_ = **6.1 S** (the value for the smallest species of HtrA S221A sample from the H. pylori N6 strain – see Table 4), and that the value of frictional ratio for all oligomers is the same.*

**FIGURE 8:**
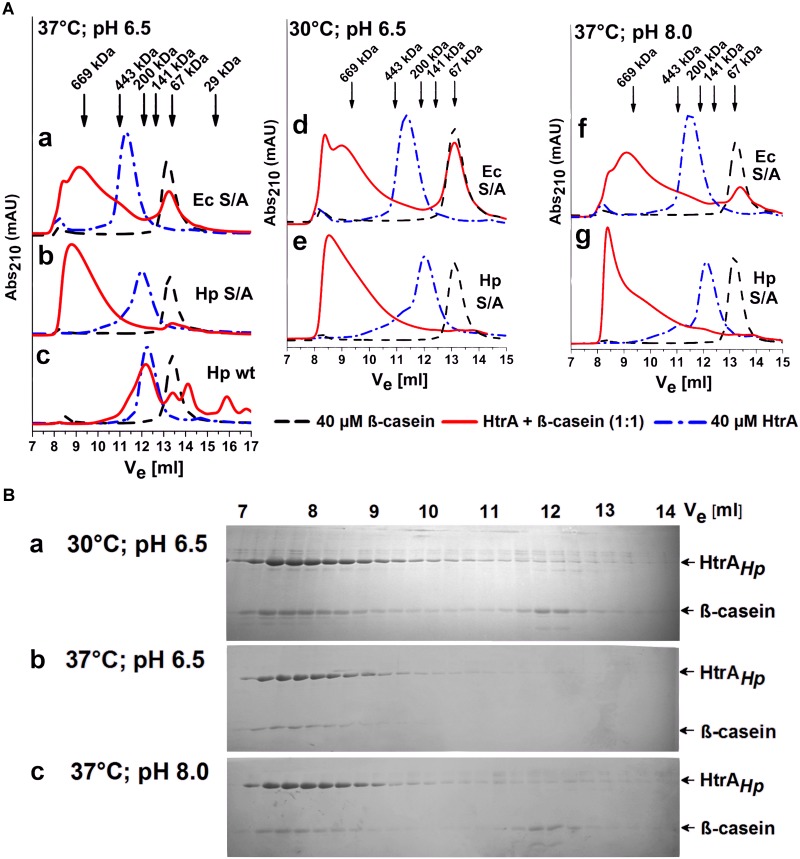
HtrA oligomer rearrangements in the presence of a substrate. **(A)** The proteolytically inactive (S/A) variants from *H. pylori* 26695 (HtrA*_Hp_*S221A) and *E. coli* (HtrA*_Ec_*S210A) were pre-incubated with β-casein at a molar ratio of 1:1 at various temperatures and pH values, as indicated, and then analyzed using size exclusion chromatography (SEC). Active wt HtrA*_Hp_* is included as a control in subpanel c. The arrows indicate the elution volumes of the molecular weight markers. V_e_ stands for the elution volume. **(B)** The protein fractions collected in the course of SEC were resolved in 15% polyacrylamide gels and stained with Coomassie Brilliant Blue.

Another structural property of certain bacterial HtrAs is their ability to rearrange oligomers upon binding of suitable substrates. For example, in the resting state, HtrA*_Ec_* forms hexamers which are converted into 12-mers or 24-mers in the presence of a substrate (Clausen et al., 2011). In many cases, these rearrangements are associated with the allosteric regulation of the protease activity (de Regt et al., 2015). To investigate if HtrA*_Hp_* undergoes such substrate-dependent remodeling, we analyzed its oligomeric forms in the presence and absence of a substrate, using size exclusion chromatography. The experiments were performed using the refolded proteolytically inactive S221A variant of the *H. pylori* strain 26695. In agreement with the sedimentation experiments, the ligand-free HtrA*_Hp_*S221A eluted predominantly in a fraction containing a mixture of trimeric and hexameric forms (between 141 and 200 kDa). A larger oligomeric form (between 200 and 443 kDa) was visible as a shoulder of the main elution peak (Figure 8A subpanels b, e, g). The control HtrA*_Ec_*S210A protein was eluted as a single fraction corresponding to a hexamer (Figure 8A subpanels a, d, f), as previously reported (Krojer et al., 2008b; Zarzecka et al., 2018). Incubation with the substrate β-casein changed the elution profile of both HtrA proteins, as oligomeric forms of approximately 700 kDa and larger were detected. At a molar enzyme/substrate ratio of 1:1 almost the entire HtrA*_Hp_*S221A pool was present as large oligomers that had complexed the majority of β-casein molecules (Figure 8A subpanels b, e, g), especially at 37°C and pH 6.5 (Figure 8B, subpanel b). As expected, HtrA*_Ec_*S210A formed high order oligomers as well, however, a significant fraction of β-casein remained unbound under the same experimental conditions (Figure 8A subpanel a, d, f). These results indicate that HtrA*_Hp_* undergoes substrate-dependent structural rearrangements, similar to those observed with HtrA*_Ec_*. Moreover, it seems that proteolytically inactive HtrA*_Hp_* binds β-casein with a higher affinity than HtrA*_Ec_*.

The HtrA*_Ec_* large oligomers that represent the active state of the protein are known to disassemble and return to a resting state once the substrate is depleted (Kim et al., 2011). Oligomers formed by HtrA*_Hp_* displayed similar properties. As judged from the elution profiles of the proteolytically active wt HtrA*_Hp_* co-incubated with β-casein, large assemblies were no longer detected once substrate degradation was completed (Figure 8A, subpanel c).

## Discussion

The aim of this work was to determine the role of *H. pylori* HtrA protease in stress protection, which may help the bacterial cell to adapt to the harsh environmental conditions in the human stomach and to establish a persistent infection in humans. Together, this work further expanded our knowledge regarding the biochemical properties of this protein. Using a recently established *H. pylori* N6 deletion mutant strain (N6 Δ*htrA*), we were able for the first time to directly examine the essentiality of HtrA*_Hp_* under stress conditions. The N6 Δ*htrA* bacteria together with the control of isogenic N6 wt and the *htrA* complementation mutant (N6 Δ*htrA/htrA_N6_*) were challenged with elevated temperatures, acidic or basic pH, oxidative stress, increased concentrations of ionic or nonionic osmotica, and the antibiotic puromycin. All of these stress factors are known to affect cellular proteostasis. Puromycin interferes with protein translation and results in premature termination of protein synthesis with release of truncated polypeptides that are potentially toxic for a cell (Azzam and Algranati, 1973). Osmotic, acid, basic, oxidative or heat stress can result in protein denaturation and possibly aggregation of subsequently misfolded proteins. In all these situations, any nonfunctional proteins must be removed to restore cellular homeostasis, mainly via proteolysis, in which a role of HtrA was anticipated. Indeed, we found that the *htrA* deletion mutant of strain N6 was markedly more susceptible to almost all stress conditions tested here, when compared to its parental wt control. The only exception was oxidative stress, and we did not observe increased sensitivity of the *H.* pylori *ΔhtrA* cells to such oxidants, as H_2_O_2_ or cumene hydroperoxide. This result was surprising, as HtrA is implicated in oxidative stress tolerance in several bacterial species (Skorko-Glonek et al., 2013). However, it is important to note that the *C. jejuni htrA* mutant is not more vulnerable to oxidants than the wt bacteria when grown at physiological temperatures (Brøndsted et al., 2005). The importance of HtrA for growth and survival of *C. jejuni* cells was demonstrated under combined stress conditions, i.e., oxidative and thermal stresses (42–44°C) (Baek et al., 2011). Unfortunately, the *H. pylori ΔhtrA* mutant is less tolerant to elevated temperatures than *C. jejuni htrA*, and our combined stress assays were carried at a lower temperature (39°C). Probably, the overall effect of these stresses on cellular proteins was not strong enough to select for the presence of HtrA*_Hp_*.

The importance of HtrA*_Hp_* for stressed bacteria proves that this protein plays a crucial protective role in the *H. pylori* cell to overcome effects of various environmental stresses. By comparison, HtrA*_Ec_* in *E. coli* is regarded as the most important protease involved in extracytoplasmic protein quality control, as the enzyme removes aberrant proteins from the cellular envelope. *In vitro*, HtrA*_Ec_* is known to preferably degrade unfolded polypeptides but not native, well-structured proteins (Skorko-Glonek et al., 2003; Skorko-Glonek et al., 2008; Jomaa et al., 2009). We propose that HtrA*_Hp_* may play a similar role in *H. pylori*. The ability of this protease to efficiently degrade unstructured proteins (e.g., β-casein) or denatured proteins (reduced lysozyme), but not properly folded proteins (native lysozyme) (Figure 4) confirms this assumption. The cleavage site specificity of HtrA*_Hp_* was determined and this was also typical for proteases involved in protein quality control. With the use of model substrates, we determined that HtrA*_Hp_* prefers hydrophobic residues (V, L, I) at the P1 position (Figure 6B–D and Table 2). These nonpolar residues are particularly exposed in unstructured or denatured proteins, while they are usually inaccessible in native, properly folded proteins. A very similar cleavage specificity at P1↓P1’ was demonstrated for the natural HtrA*_Hp_* substrate, E-cadherin. This human target protein was preferentially cleaved at the consensus motif [VITA]↓[VITA]-x-x-D-[DN] (Schmidt et al., 2016). However, the model substrates used here did not reveal any preference for amino acid sequences more distant from the cleavage site. Nevertheless, nonprime cleavage sequence determinants most probably play an important role in choosing the cutting site by HtrA*_Hp_*. Although a similar preference by HtrA*_Hp_* and HtrA*_Ec_* was observed for residues at P1↓P1’, the proteases produced different β-casein or lysozyme degradation products (Figure 6A). The sequences flanking the active site region, including residues known to form the S1 specificity pocket responsible for selective binding of a side chain of amino acid at P1, are well conserved in both proteins. According to the crystal structure of HtrA*_Ec_*,*_,_* the S1 specificity pocket is composed of I205 (the L1 loop), A227, and I228 (the L2 loop) (Krojer et al., 2010). In HtrA*_Hp_*, the corresponding residues are I216 (L1), A238, and I239 (L2), hence the identical amino acids (Supplementary Figure S4). This observation supports the assumption that the different substrate cleavage products produced by HtrA*_Hp_* and HtrA*_Ec_* must be caused by different interactions at the sites distant from the active centers of these enzymes.

In this study, HtrA*_Hp_* was shown to be very tolerant to a wide range of temperatures and pH values. Previous work demonstrated that this protease can degrade E-cadherin, one of its natural substrates during infection, at pH 4–10 and at temperatures up to 65°C (Hoy et al., 2012). Here, we expanded the characteristics of proteolytical properties of HtrA*_Hp_* toward the model substrate β-casein, at various temperatures and pH values. β-casein was degraded much faster than E-cadherin (minutes versus hours, respectively) and this allowed us to determine the apparent cleavage rates for this substrate. We found that HtrA*_Hp_* is distinguished among other HtrA homologs by its ability to operate under extreme, nonphysiological temperature conditions, as high as 80°C, with the maximum at 75°C (Figure 5B). In contrast, HtrA*_Ec_* was inactivated at these temperatures. The HtrA homologs from other pathogenic bacteria described thus far are characterized with lower optimal temperatures for catalysis. For example, HtrA from *C. jejuni* prefers temperatures of about 50°C (Baek et al., 2011) and that of *Edwardsiella tarda* 40°C (Jiao et al., 2010), whereas the homologs derived from environmental species work best at even lower temperatures (*Synechocystis* sp. 35°C, *Stenotrophomonas maltophilia* 35–37°C) (Huesgen et al., 2011; Zarzecka et al., 2018).

The HtrA*_Hp_* pH dependence profile was similar but not fully overlapping to that of HtrA*_Ec_*. HtrA*_Hp_* was highly active at all tested pH values and exhibited its maximal activity at around pH 6.5, whereas HtrA*_Ec_* preferred lower values with an optimum at pH 5.5 and its activity was low when the pH exceeded 8.0 (Figure 5A). Generally, HtrA*_Hp_* was more active than HtrA*_Ec_* under most studied conditions. The T_m_ of HtrA*_Hp_* (above 85°C) confirmed the exceptional stability of this protein (Table 3), while T_m_ values of previously examined HtrA homologs were markedly lower: 67.5–73°C for HtrA*_Ec_* and 56–61°C for *S. maltophilia* (Zarzecka et al., 2018). While analyzing the results of casein zymography, we noticed that part of the HtrA*_Hp_* preparation migrated slower in the gel, suggesting the presence of trimers or other oligomers (Figure 3). These results imply that formed HtrA*_Hp_* oligomers are remarkably stable and can withstand the denaturing conditions of gel electrophoresis. Interestingly, the HtrA*_Hp_* proteins produced from three different *H. pylori* strains maintained different pools of oligomers with two distinct patterns to be observed: (i) a predominance of monomers was seen for the protein from strain 26695 and (ii) a high content of oligomeric forms was obtained for strains J99 and N6 (Figure 3). The observed strain-specific differences in HtrA*_Hp_* oligomer assembly and stability were confirmed with higher precision by sedimentation velocity ultracentrifugation. HtrA*_Hp_* from strain 26695 was detected predominantly as a mixture of trimers, hexamers and nonamers in dynamic equilibrium. To the contrary, HtrA*_Hp_* from the strain N6 was characterized by the presence of stable trimers and a higher content of large and stable oligomers (mainly 18-mers) (Figure 7 and Table 4). The sequences of HtrA*_Hp_* from strains 26695 and N6 are 99% identical and differ only by five amino acids (Supplementary Figure S4). We can only speculate that these strain-specific substitutions are responsible for the observed differences in stability of the HtrA*_Hp_* oligomers. However, this assumption requires further experimental confirmation and is currently under investigation.

## Conclusion

In this work, we present the effects of deleting the *htrA_Hp_* gene of *H. pylori* in cell physiology and stress survival. We found that bacteria deprived of HtrA*_Hp_* are much more sensitive to a variety of experimental stress conditions. These results and the biochemical properties of the HtrA*_Hp_* protein as determined here indicate the function of this protein is mainly in the protein quality control. HtrA*_Hp_* is exceptionally resistant to temperature and pH variations, and has the capability to regain activity following treatment at denaturing conditions, which indicates that HtrA*_Hp_* is well adapted to operate in harsh conditions, either in the form of exported protein or to maintain protein functionality in the periplasmic space. Thus, HtrA*_Hp_* should be regarded as a major player of this important bacterial pathogen to colonize and persist in the human stomach for a life time.

## Author Contributions

JS-G, UZ, and SB conceptualized the study. UZ, AM-W, AL, DF, and MA performed the experiments and generated the data. JS-G, SB, UZ, AZ-P, AM-W, AB, and BL analyzed and interpreted the data. JS-G and UZ wrote the manuscript. All authors revised and agreed on the manuscript.

## Conflict of Interest Statement

The authors declare that the research was conducted in the absence of any commercial or financial relationships that could be construed as a potential conflict of interest.
