# Supplementary material for: Properties of the HtrA Protease From Bacterium Helicobacter pylori Whose Activity Is Indispensable for Growth Under Stress Conditions
Source: Front Microbiol. 2019 May 3;10:961. doi: 10.3389/fmicb.2019.00961 (PMC6509562; doi:10.3389/fmicb.2019.00961)
Supplement: Supplementary file 1 [file Data_Sheet_1.pdf]

Supplementary Material

Properties of the HtrA protease from bacterium *Helicobacter pylori* whose activity is indispensable for growth under stress conditions

Urszula Zarzecka, Anna Modrak-Wójcik, Donata Figaj, Malgorzata Apanowicz, Adam Lesner, Agnieszka Bzowska, Barbara Lipinska, Anna Zawilak-Pawlik, Steffen Backert and Joanna Skorko-Glonek*

*** Correspondence:** J.Skorko-Glonek joanna.skorko-glonek@biol.ug.edu.pl

Supplementary Table S1. PCR primers used in the work.

	**Orientations**	**Sequence (5`> 3`)**
HphtrA_rev	reverse	ATACTCGAGTTTCACCAAAATGATTCTATAACCTTG
HphtrAJ99_fw	forward	ATACCATGGGCAATATCCAAATCCAAAACATGCCC
HphtrA26695_fw	forward	ATACCATGGGCAATATCCAAATCCAGAGCATGCCC
Hp_S221A_fw	forward	CCTGGAAATGCCGGCGGCGCTTTAATTGATAGC
Hp_S221A_rev	reverse	GCTATCAATTAAAGCGCCGCCGGCATTTCCAGG

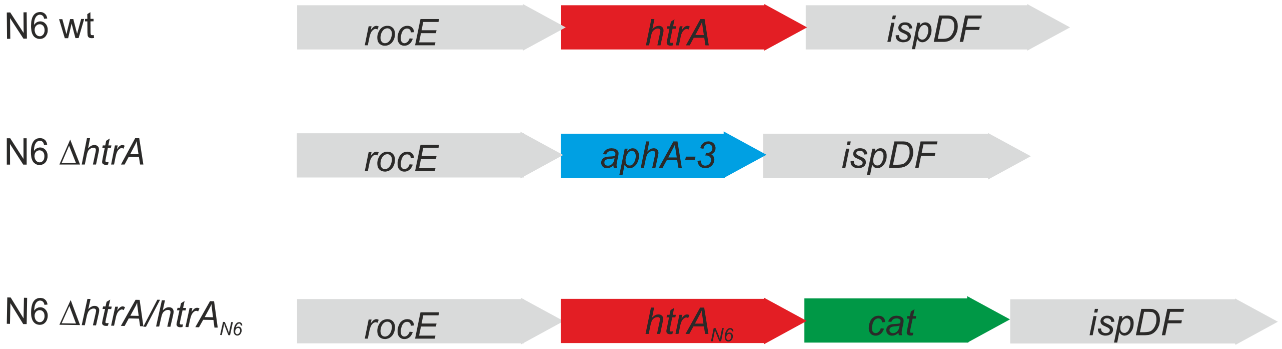

Supplementary Figure S1. Schemes of the *htrA* locus and the flanking *H. pylori* chromosomal regions in the wt, Δ*htrA*, and Δ*htrA*/*htrA* complemented N6 strains

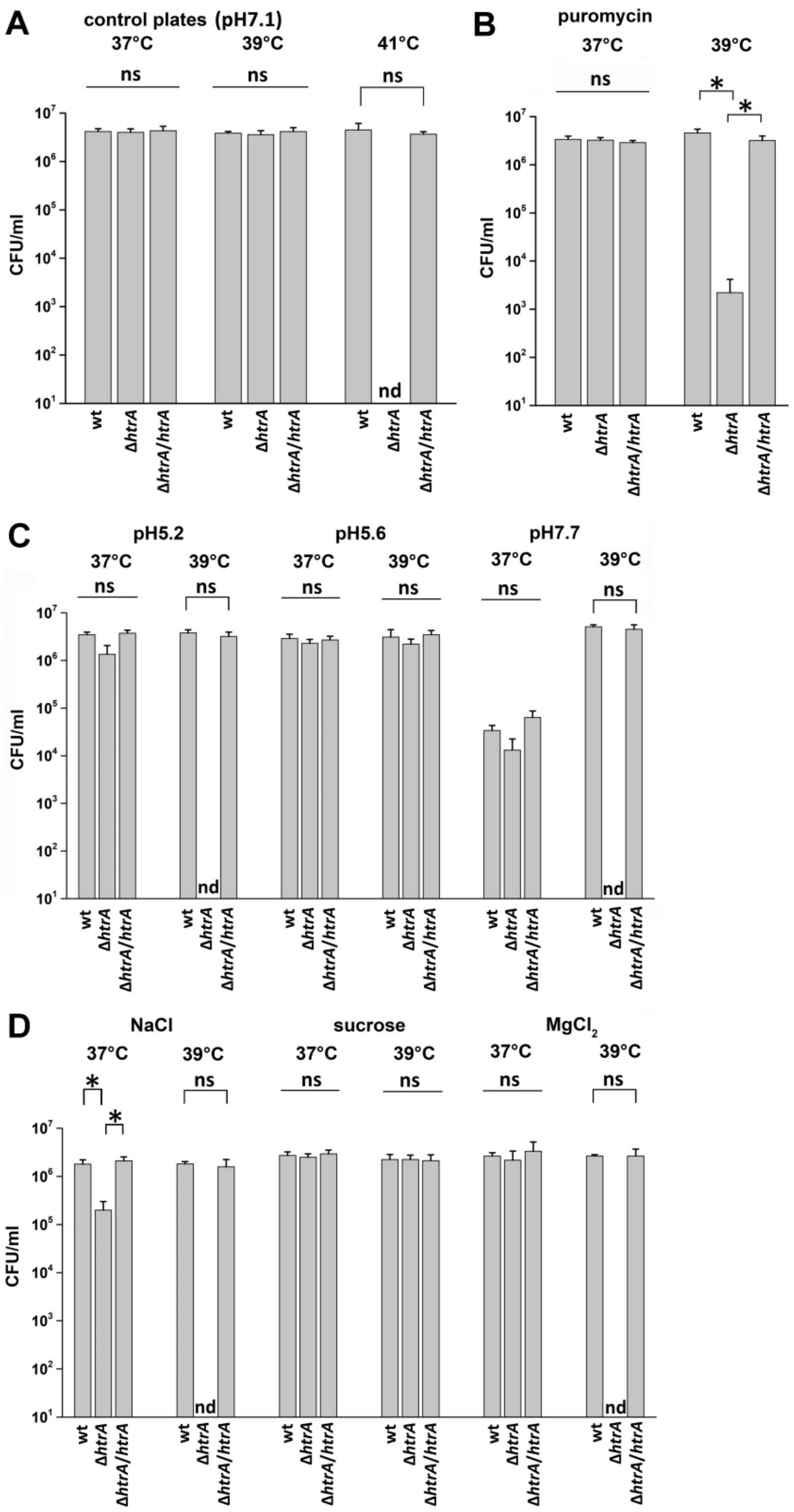

Supplementary Figure S2. Survival of the stress-exposed *H. pylori* cells. The standard error of mean (SEM) was calculated using at least three repetitions. Data were evaluated using Bonferroni test. Statistical significance was defined by $p \leq 0.05$ (*). nd- no single colonies detected; ns- no significant differences. The representative pictures presenting *H. pylori* growth under the analyzed stress conditions are presented in Figures 1A, B, 2A, and B that correspond to parts A, B, C and D of this figure, respectively.

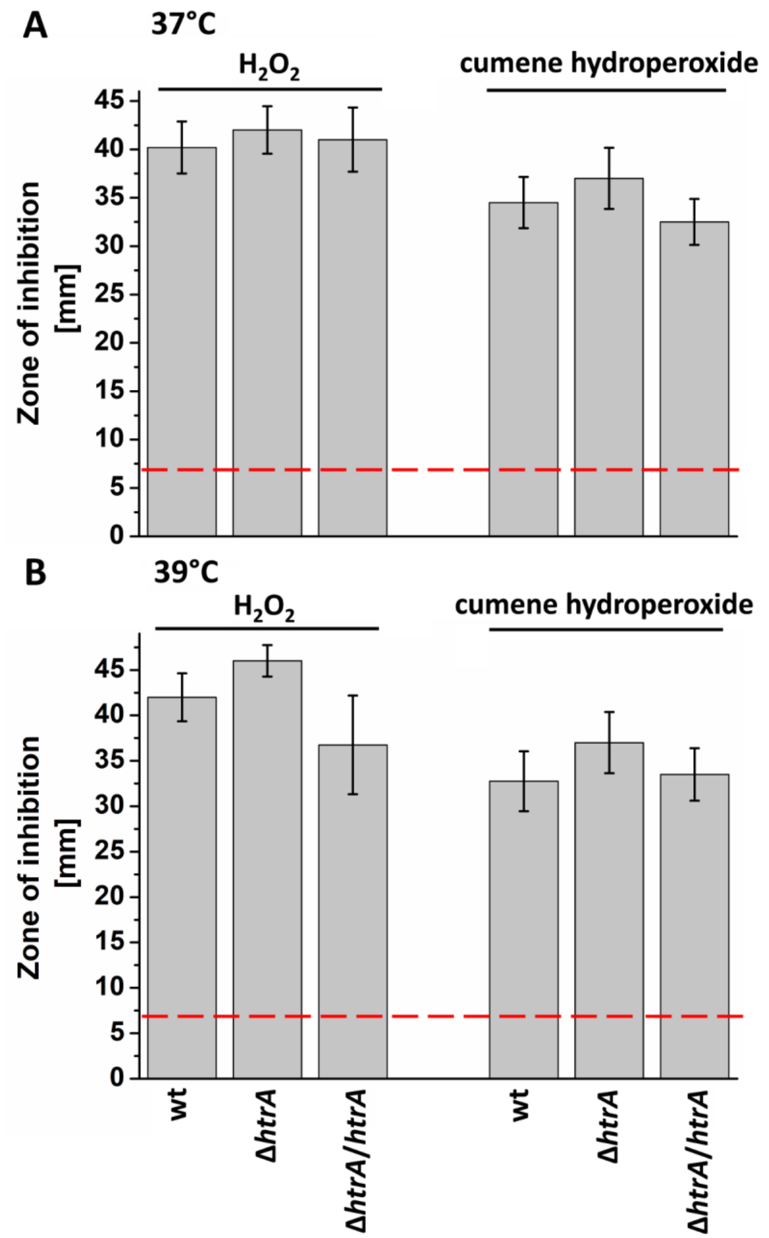

Supplementary Figure S3. Effects of oxidative stress on growth of *H. pylori* N6 Δ*htrA*. Bacterial cultures of the N6 Δ*htrA* and the control strains (N6 and *htrA* complementation) were cultured and challenged with H_2O_2 and cumene hydroperoxide as described in the Materials and Methods section. Plates were incubated for 3 days at (A) 37°C and (B) 39°C. The experiments were performed at least three times. The red dotted line indicates the diameter of the filter disc. Analysis by Bonferroni test showed no significant differences ($p < 0.05$) between the tested strains for all experimental conditions.

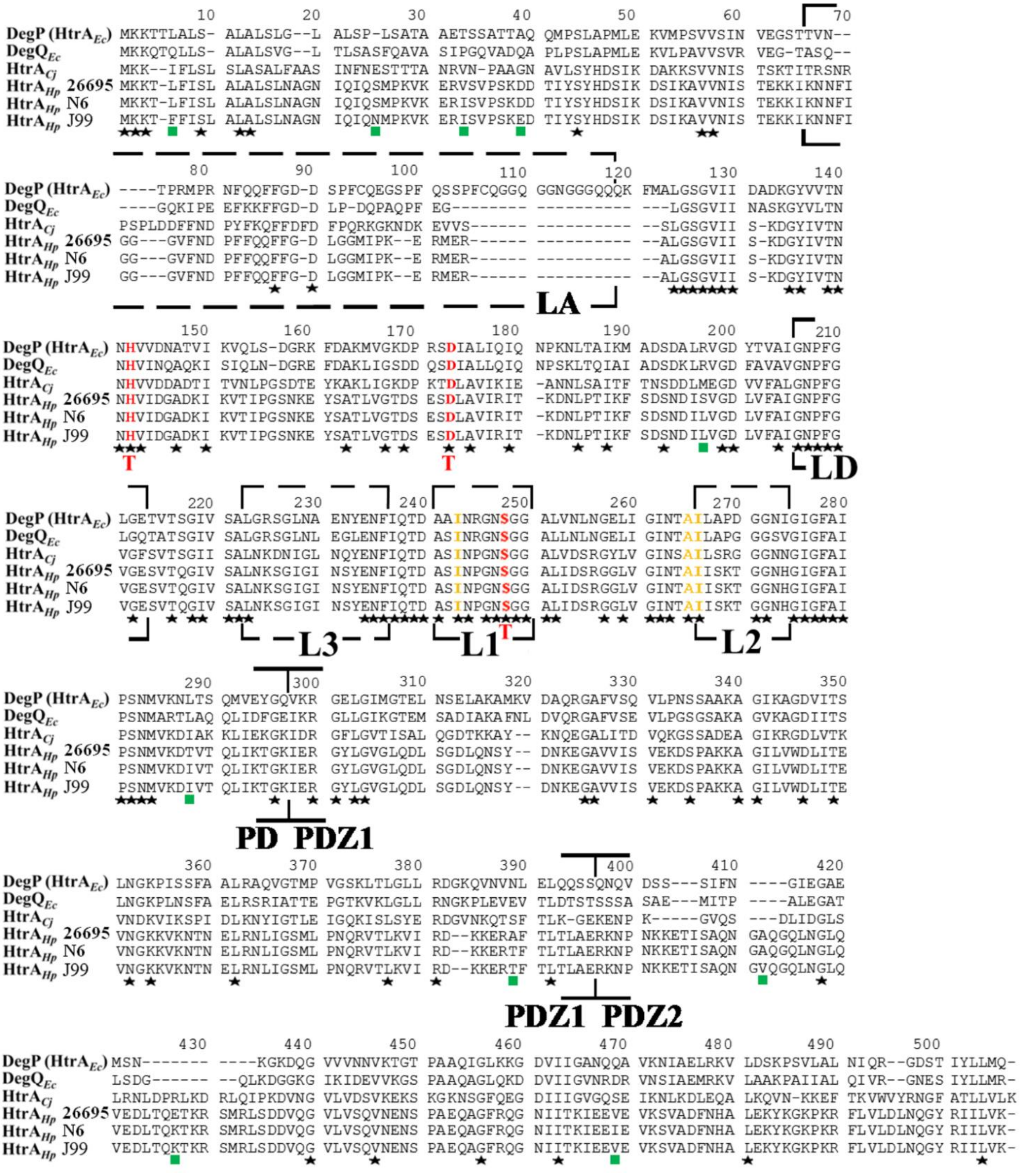

Supplementary Figure S4 Comparison of the amino acid sequences of the selected HtrA homologs: HtrA (DegP) and DegQ *E. coli*, HtrA *C. jejuni* and HtrA *H. pylori* (26695, N6 and J99 strains). The important regulatory loops (LA, LD, L1, L2, L3) as well as domain organisation are marked with dashed lines. PD-stands for the protease domain, T- catalytic triad. Asterisks indicate the conserved residues. The S1 specificity pocket is marked in orange within the L1 and L2 loops. Green squares indicate differences between $HtrA_{Hp}$ from *H. pylori* 26695, N6 and J99 strains.

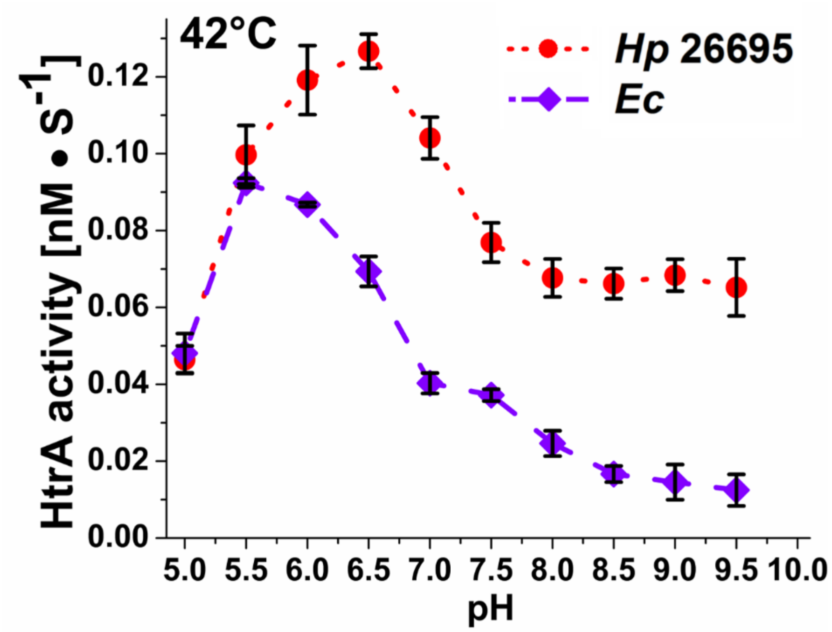

Supplementary Figure S5. pH-dependence of the $HtrA_{Hp}$ (*Hp* 26695) and $HtrA_{Ec}$ (*Ec*) at 42°C. The error bars represent the standard deviation values from three independent measurements.
